# Large‐Scale Proteome Profiling Identifies Biomarkers Associated with Suspected Neurosyphilis Diagnosis

**DOI:** 10.1002/advs.202307744

**Published:** 2024-02-21

**Authors:** Jun Li, Jie Ma, MingJuan Liu, Mansheng Li, Ming Zhang, Wenhao Yin, Mengyin Wu, Xiao Li, Qiyu Zhang, Hanlin Zhang, Heyi Zheng, Chenhui Mao, Jian Sun, Wenze Wang, Wei Lyu, Xueping Yue, Wenjia Weng, Juan Li, Fengxin Chen, Yunping Zhu, Ling Leng

**Affiliations:** ^1^ Department of Dermatology Institute of Clinical Medicine State Key Laboratory of Complex Severe and Rare Diseases Peking Union Medical College Hospital Chinese Academy of Medical Sciences and Peking Union Medical College National Clinical Research Center for Dermatologic and Immunologic Diseases Beijing 100730 China; ^2^ Stem cell and Regenerative Medicine Lab Department of Medical Science Research Center Institute of Clinical Medicine State Key Laboratory of Complex Severe and Rare Diseases Translational Medicine Center Peking Union Medical College Hospital Peking Union Medical College and Chinese Academy of Medical Sciences Beijing 100730 China; ^3^ State Key Laboratory of Medical Proteomics Beijing Proteome Research Center National Center for Protein Sciences (Beijing) Beijing Institute of Lifeomics Beijing 102206 China; ^4^ Department of Dermatology Beijing Youan Hospital Capital Medical University Beijing 100069 China; ^5^ The First Hospital of Jiaxing The Affiliated Hospital of Jiaxing University Zhejiang 314001 China; ^6^ Department of Neurology Peking Union Medical College Hospital Peking Union Medical College and Chinese Academy of Medical Science Beijing 100730 China; ^7^ Department of Pathology Peking Union Medical College Hospital Chinese Academy of Medical Sciences and Peking Union Medical College Beijing 100730 China; ^8^ Department of Infectious Disease Peking Union Medical College Hospital Chinese Academy of Medical Sciences and Peking Union Medical College Beijing 100730 China; ^9^ Department of Dermatology and Venereology Beijing Tiantan Hospital Capital Medical University Beijing 100050 China; ^10^ Infections Disease Center Beijing Ditan Hospital Capital Medical University Beijing 100102 China; ^11^ Basic Medical School Anhui Medical University Anhui 230032 China

**Keywords:** cerebrospinal fluid, diagnostic biomarker, machine learning model, neurosyphilis, proteomics

## Abstract

Neurosyphilis (NS) is a central nervous system (CNS) infection caused by *Treponema pallidum* (*T. pallidum*). NS can occur at any stage of syphilis and manifests as a broad spectrum of clinical symptoms. Often referred to as “the great imitator,” NS can be easily overlooked or misdiagnosed due to the absence of standard diagnostic tests, potentially leading to severe and irreversible organ dysfunction. In this study, proteomic and machine learning model techniques are used to characterize 223 cerebrospinal fluid (CSF) samples to identify diagnostic markers of NS and provide insights into the underlying mechanisms of the associated inflammatory responses. Three biomarkers (SEMA7A, SERPINA3, and ITIH4) are validated as contributors to NS diagnosis through multicenter verification of an additional 115 CSF samples. We anticipate that the identified biomarkers will become effective tools for assisting in diagnosis of NS. Our insights into NS pathogenesis in brain tissue may inform therapeutic strategies and drug discoveries for NS patients.

## Graphical abstract

This study presents an innovative approach to diagnosing Neurosyphilis (NS), a complex central nervous system (CNS) infection. By analyzing 223 cerebrospinal fluid samples using proteomic techniques and machine learning models, researchers identifies three key biomarkers (SEMA7A, SERPINA3, and ITIH4) for NS. These findings, validated through multicenter research with additional 115 samples, offer new diagnostic tools and deepen understanding of NS pathogenesis.

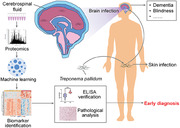

## 1 Introduction

Syphilis remains a public health concern in the 21st century,^[^ ^1^ ^]^ and the recent resurgence of syphilis in China has coincided with an increase in neurosyphilis (NS) cases.^[^ ^2^ ^]^ Previous studies have shown that the detection rate of *Treponema pallidum (T. pallidum)* in the cerebrospinal fluid (CSF) of patients with untreated syphilis (both primary and secondary) can reach 40%.^[^ ^3^, ^4^, ^5^ ^]^ Early infection may manifest as asymptomatic meningitis and lead to severe organ damage and irreversible dysfunction and even become life‐threatening if left untreated.^[^ ^5^ ^]^ Therefore, early identification and treatment of NS are of paramount importance. However, none of the existing tests can be considered an applicable gold standard, and no consensus on diagnostic criteria for NS has been reached.

Current diagnostic criteria for NS are mainly based on comprehensive consideration of clinical manifestations and CSF tests (CSF cell count or protein and a reactive CSF‐Venereal Disease Research Laboratory [VDRL] or rapid plasma reagin [RPR]) test. According to the diagnostic criteria published by the Centers for Disease Control and Prevention (CDC) USA, NS can be categorized into two types: (a) “confirmed” NS, as defined as a positive VDRL/RPR test in CSF at any stage of syphilis; and (b) “presumptive” neurosyphilis, as defined as CSF‐VDRL/RPR test negative but with an elevated CSF cell count and/or protein and clinical symptoms or signs consistent with NS without other known causes.^[^ ^6^ ^]^ Although the specificity of CSF‐VDRL test detection is high, its sensitivity is relatively low (10%–70%),^[^ ^7^, ^8^, ^9^, ^10^ ^]^ leading to a high rate of false negatives and misdiagnosis of NS. This issue imposes a significant health and economic burden on patients and causes great consumption of healthcare resources. Overall, exploration of disease‐specific biomarkers is critical for understanding the pathogenesis of NS, improving the early diagnosis rate, and reducing the incidence of complications.

In recent years, significant strides in research of central nervous system diseases, such as neurodegenerative diseases, CNS trauma, tumors, and infections, have been achieved through proteomics technology.^[^ ^11^, ^12^, ^13^, ^14^, ^15^ ^]^ However, there are no reports of CSF proteomics for NS patients. In this study, we applied mass spectrometry (MS)‐based proteomics and verification experiments to 338 CSF samples (Table S1, Supporting Information) collected from patients with NS, posttreatment NS (PTNS), or syphilis/non‐NS (NNS), infectious brain disease unrelated to syphilis (IBD), and noninfectious brain disease (NIBD) without syphilis. Our goal was to gain a better understanding of proteomic changes in NS patients and identify potential biomarkers for early NS diagnosis.

## 2 Results

### 2.1 Proteomic Features of CSF from NS Patients

**Figure 1:**
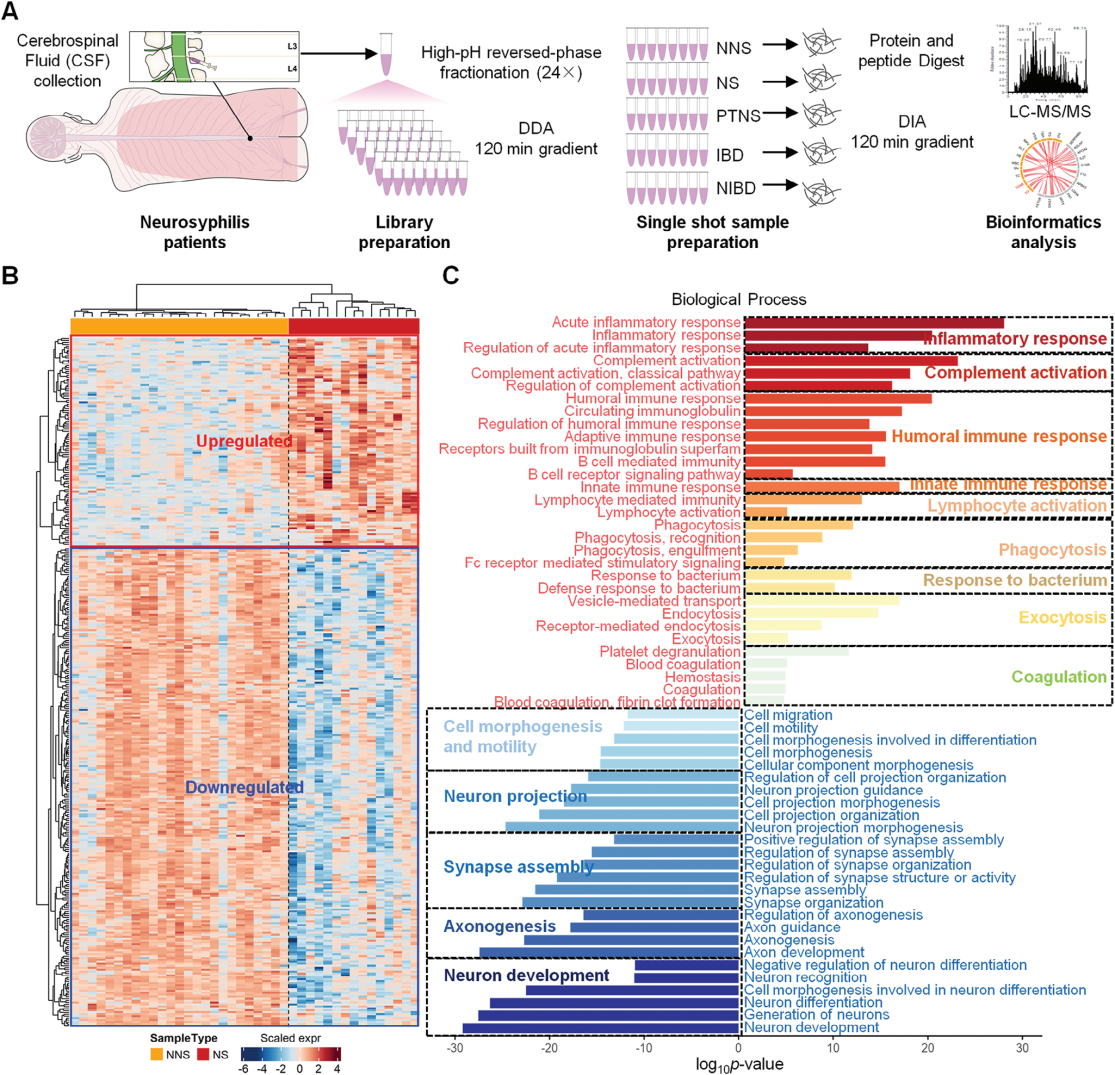
Proteomic features of CSF from NS patients. A) Schematic of the experimental workflow of the quantitative proteome and bioinformatics analyses used to analyze CSF samples from NNS (*n* = 25), NS (*n* = 15), PTNS (*n* = 7), NIBD (*n* = 9), and IBD (*n* = 7) patients. High‐resolution MS analyses of fractionated, pooled samples were performed in data‐dependent acquisition mode (DDA) for library construction and subsequently in data‐independent (DIA) acquisition mode for protein identification and quantitation. NNS: syphilis/nonneurosyphilis; NS: neurosyphilis; PTNS: posttreatment neurosyphilis; NIBD: noninfectious brain disease without syphilis; IBD: infectious brain disease unrelated to syphilis. B) Heatmap showing differentially expressed proteins (358 proteins in total) in the CSF between NNS (*n* = 25) and NS (*n* = 15) samples (Cohort 1). Pairwise comparisons are carried out using Limma and the proteins with a Benjamini–Hochberg (BH) adjusted *p* value ≤ 0.01 were considered to indicate statistical significance. The red and blue empty frames or boxes represent up‐ and downregulated proteins, respectively, in the NS vs. NNS comparison. C) Biological process analysis of differentially expressed proteins in the CSF of NS vs. NNS patients ranked according to the log10 *p* value. The colors indicate functional categories. Changes in expression levels of selected functional proteins that were significantly upregulated (red font) or downregulated (blue font) between NNS and NS samples.

We collected 63 CSF samples from 15 NS patients, 7 PTNS patients, 9 NIBD patients, 7 IBD patients, and 25 NNS patients for proteome identification using a data‐independent acquisition (DIA) strategy (**Figure** **1**A and Table S1, Supporting Information). In total, 1800 proteins were identified, including 1749 from NS, 1638 from PTNS, 1638 from NIBD, 1549 from IBD, and 1719 from NNS patients (Table S2; Figure S1A, Supporting Information). The results showed good correlations of protein abundance within samples belonging to the NS and NNS groups (Cohort 1) (Figure S1B, Supporting Information). However, correlations between the NS and NNS groups were poor. Principal coordinate analysis (PCoA) was used to quantify the dissimilarity of NS and NNS groups (Figure S1C, Supporting Information). CSF‐RPR (RPRs high; RPR titer > 1:4), CSF‐white blood cell (WBCs; WBC high; WBC > 8×10^6^ cells L^−1^), and CSF‐protein (PROs; PROhigh; PRO > 0.45 g L^−1^) levels in the CSF of NS patients were greater than those in the CSF of NNS patients (Figure S1D, Supporting Information). Hierarchical clustering analysis (HCA) and volcano map analysis revealed 358 proteins differentially expressed between the NS and NNS groups (Figure 1B; Table S2, Figure S2A, Supporting Information). The 109 upregulated proteins in the NS group were predominantly enriched in the immune response, including innate and humoral immunity, as well as various inflammatory responses (Figure 1C), compared to those in the NNS group. We also found upregulated expression of proteins associated with the defense response to bacteria, coagulation, and phagocytosis in the NS group, suggesting the potential contribution of various wound healing‐associated proteins in the CSF. Proteins with downregulated expression in NS patients were predominantly associated with nervous system development, including axonogenesis, synapse organization, and neuron development (Figure 1C). Kyoto Encyclopedia of Genes and Genomes (KEGG) pathway analysis revealed that downregulated proteins, such as NOTCH1/2 and Netrin‐1, in the NS group are involved in nervous system development (Figure S2B, Supporting Information).

### 2.2 Potential Biomarkers Distinguishing NS from NNS Patients

**Figure 2:**
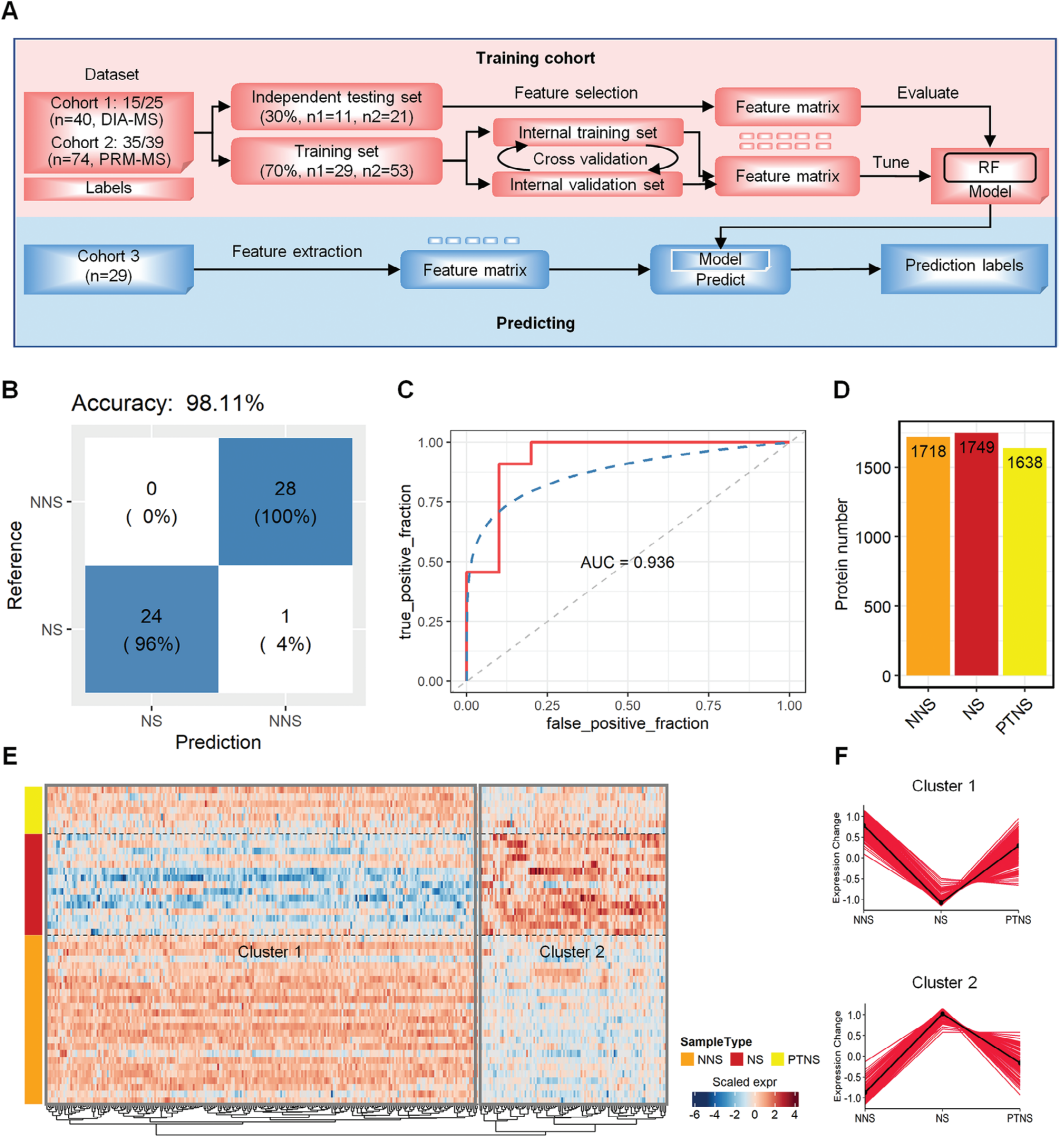
Identification of potential biomarkers for diagnosis of NS patients from NNS patients using machine learning methods. A) Schematic of the random forest (RF)‐based machine learning strategy developed for classifying the CSF of NS patients. The classifier was first trained and validated in two training and independent testing cohorts (Cohort 1 and Cohort 2) and then validated in a third independent testing cohort (Cohort 3). Cohort 1 included protein profiles from a cohort consisting of 15 NS individuals and 25 NNS individuals quantified via DIA‐MS. Cohort 2 was a protein profile of 35 NS individuals and 39 NNS individuals verified via PRM‐MS analysis. Cohort 3 included the protein profiles of 29 individuals suspected of having NS detected via PRM‐MS analysis, and 11 of them were verified via ELISA after prediction via the RF model. B) Confusion matrix showing the model performance for classifying NS individuals. The numbers represent the total number of repeats from three cross‐validations with train‐test splits. C) Receiver operating characteristic curves for the RF‐based model for classifying NS individuals. The red line shows values for the test cohort. Random performance is indicated by the gray dotted diagonal line. D) Distribution of proteins identified in CSF from NNS, NS, and PTNS samples. E) Heatmap showing the changes in expression of differentially expressed proteins (358 total proteins, z score‐normalized log2‐transformed value) in the NNS and NS samples within the PTNS sample. The red and blue boxes represent up‐ and downregulated proteins, respectively, among the three groups. F) Coexpression patterns of the proteins in the Cluster 1 and Cluster 2 modules are shown, representing proteins that were upregulated (cluster 1) and downregulated (cluster 2) in the NS group compared to the NNS group and recovered in the PTNS group.

In general, proteins differentially expressed between the NS and NNS groups might serve as distinctive markers. To determine the importance of these characteristic proteins in identifying NS, we constructed an random forest (RF) machine learning model based on the differential proteome data from 25 NS and 15 NNS samples (Cohort 1, **Figure** **2**A). We also regenerated these RF‐based classifiers from the parallel reaction monitoring‐mass spectrometry (PRM‐MS) dataset to verify the candidate biomarkers and classify NS patients (Cohort 2, Figure 2A). This model reached an accuracy of 98.11% (Figure 2B). An area under the curve (ROC) of 0.936 was observed in the training cohort (Figure 2C). We then further tested the model on an independent cohort of 29 suspicious NS (SNS) patients (Cohort 3, Figure 2A).

Furthermore, we collected 7 PTNS patient samples and identified 1638 proteins (Figure 2D). The results revealed 232 proteins that were differentially expressed between the NS and NNS groups and were inversely expressed in CSF in the PTNS group after treatment (Figure 2E; Table S2, Supporting Information); upregulated proteins and downregulated proteins in the NS group decreased and increased, respectively, after treatment (Figure 2F). These findings suggest that these 232 differentially expressed proteins are potential biomarkers that determine the treatment efficacy and degree of recovery of NS patients after treatment.

### 2.3 Potential Biomarkers Distinguishing NS from Other Brain Diseases

**Figure 3:**
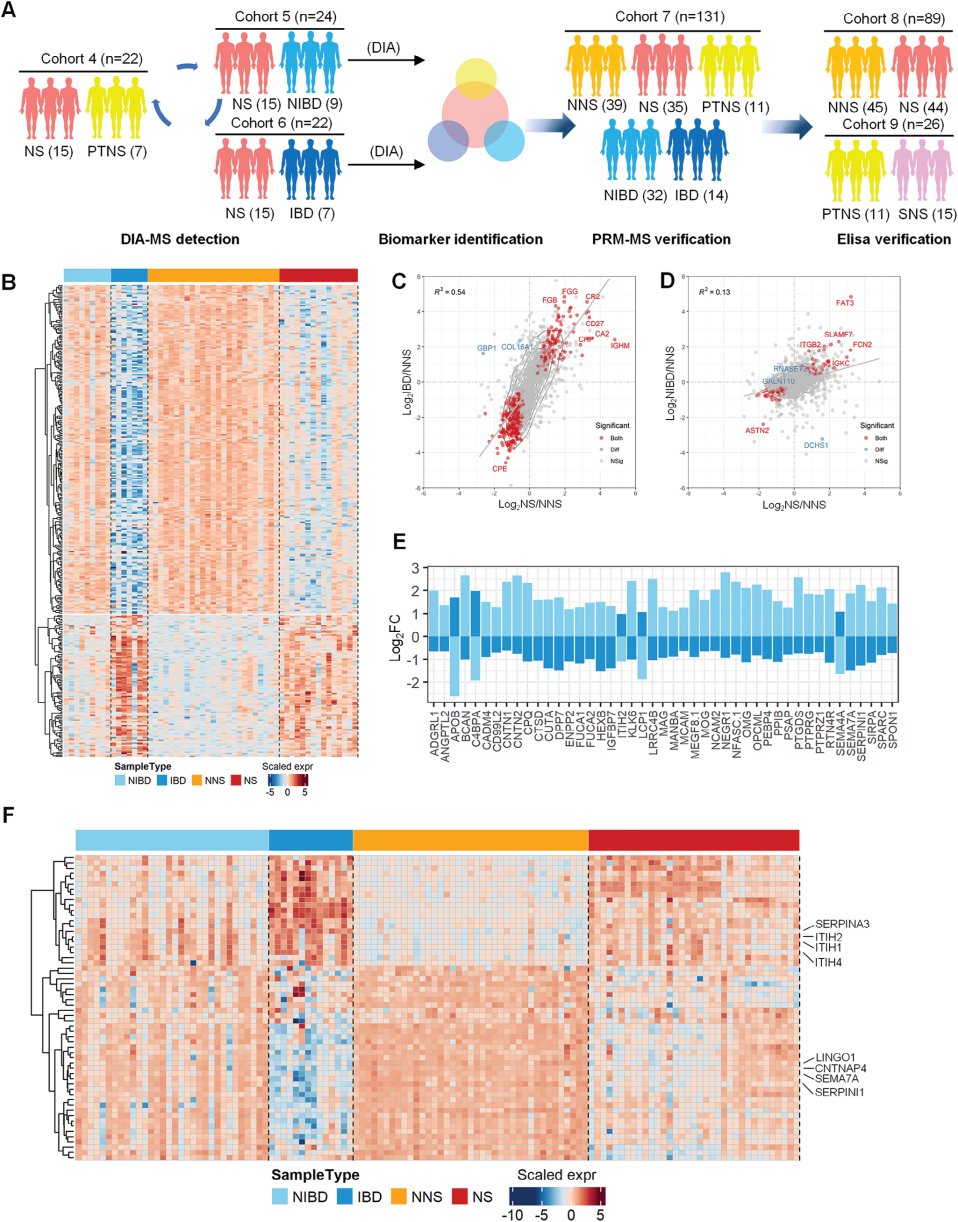
Exploration and identification of potential biomarkers for diagnosis of NS from other brain diseases. A) Workflows of CSF samples from NS, IBD, NIBD, and PTNS patients were measured by DIA‐MS, PRM‐MS, and ELISA. CSF samples were analyzed for biomarker identification based on the different cohorts. B) Heatmap showing the ability of differentially expressed proteins between NNS and NS (358 total proteins, z score‐normalized log2‐transformed value) in CSF among IBD and NIBD samples. The red and blue color bars represent the scaled expression levels of proteins among the four groups. C) Scatter plot of log2‐fold changes for NS vs. NNS (*x*‐axis) and IBD vs. NNS (*y*‐axis). The red dots indicate proteins with fold changes consistent and *p* values that met the cutoff of *p* < 0.01 for both pairwise comparisons. The blue dots indicate proteins that met the opposite fold changes and a *p* value cutoff of *p* < 0.01 for both pairwise comparisons. D) Scatter plot of log2‐fold changes for NS vs. NNS (*x*‐axis) and NIBD vs. NS (*y*‐axis). The red dots indicate proteins with consistent fold changes and *p* values that met the cutoff of *p* < 0.01 for both pairwise comparisons. The blue dots indicate proteins that met the opposite fold changes and a *p* value cutoff of *p* < 0.01 for both pairwise comparisons. E) Histogram showing differentially expressed proteins in both the NS vs. IBD (light blue) and NS vs. NIBD (dark blue) groups based on the log fold change. F) Heatmap showing differentially expressed proteins (58 proteins in total, *z* score‐normalized log2‐transformed value) in CSF among NNS (*n* = 39), NS (*n* = 35), IBD (*n* = 32), and NIBD (*n* = 14) samples for PRM‐MS verification. The red and blue color bars represent the scaled expression levels of proteins among these groups.

To further investigate whether the 358 proteins differentially expressed between the NS and NNS groups are able to distinguish NS from other brain diseases, we collected CSF samples from 7 patients with IBD (Cohort 5) and 9 patients with NIBD (Cohort 6) for quantitative proteomics (**Figure** **3**A; Figure S3A, Supporting Information). PCoA analysis revealed that most NS samples were distinguishable from those in the IBD and NIBD groups; however, small overlaps (Figure S3B, Supporting Information) indicated that the protein characteristics of NS patients were partially similar to those of IBD or NIBD patients. HCA also revealed that the protein expression patterns of NS and IBD patients were more similar (Figure 3B). Acute inflammation‐related proteins, such as CA2, IGHM, CRP, and CD27, were coexpressed (Figure 3C), suggesting the presence of strong acute response proteins in the CSF of patients with NS or other infectious brain diseases, which aligns with clinical findings. In contrast, NS patients exhibited less similarity in protein coexpression with NIBD patients (*R*2 = 0.13; Figure 3D) than with IBD patients (*R*2 = 0.54; Figure 3C). Through comprehensive analysis of 358 proteins differentially expressed between the NS and NNS groups, we identified 127 proteins that set NS apart from IBD (Figure S3C, Supporting Information) and 138 that differentiated NS from NIBD (Figure S3D, Supporting Information). Finally, we identified 45 proteins that could distinguish NS from IBD and NIBD (Figure 3E). For instance, proteins implicated in the formation of axonal connections and guidance of neuronal axons were detected. These include members of the immunoglobulin superfamily (CNTN1, CNTN2, and NCAM2), the L1 family immunoglobulin cell adhesion molecule (NFASC), semaphorin family members (SEMA4A and SEMA7A), a serpin superfamily member (SERPINI1), and a cell adhesion protein (SPON1). The analysis also revealed the presence of proteins associated with myelination, including myelin glycoprotein (MAG, MOG, and OMG), oligodendrocyte function‐associated proteins, prostaglandin D2 synthase (PTGDS), protein tyrosine phosphatase receptor type Z1 (PTPRZ1) and reticulon 4 receptor (PTN4R), in the developing nervous system (Figure 3E). These biomarkers may be instrumental in distinguishing NS from other brain diseases that are implicated in various aspects of brain function.

### 2.4 Biomarker Verification Through PRM‐MS and ELISA

**Figure 4:**
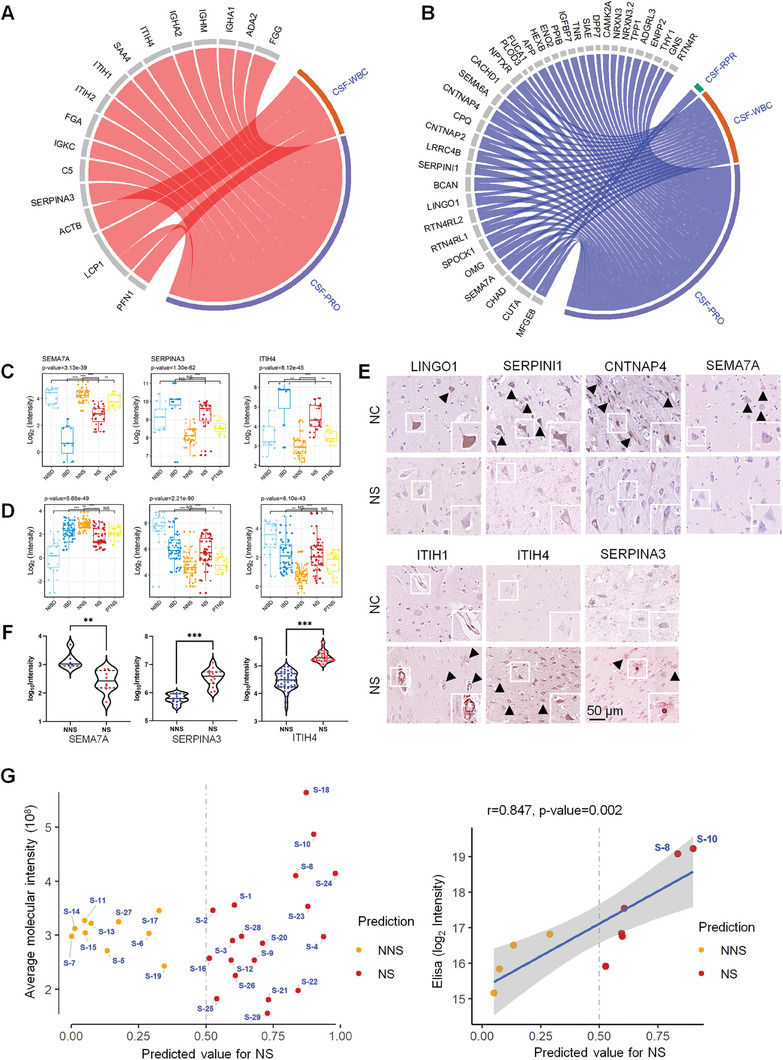
Verification of potential biomarkers for diagnosis of NS. The correlation of upregulated (A) and downregulated (B) proteins with clinical indicators (CSF‐PRP, CSF‐WBC, and CSF‐PRP). Protein expression levels of SEMA7A, SERPINA3, and ITIH4 in the NNS, NS, PTNS, IBD, and NIBD groups measured by the DIA identification (C) and PRM‐MS verification (D) according to the normalized protein intensity. Pairwise comparisons are carried out using Limma to determine the proteins with significantly different expression levels. BH‐adjusted *p* values: *p < 0.05; **p < 0.01; ***p < 0.001. E) Immunohistochemical staining of LINGO1, SERPINI1, CNTNAP4, SEMA7A, ITIH1, ITIH4, and SERPINA3 in the brain tissue of the NNS and normal control (NC) groups (scale bar: 50 µm). F) ELISA analysis of SEMA7A, SERPINA3, and ITIH4 expression in the CSF of the NNS (*n* = 45) and NS (*n* = 44) groups according to log10 (intensity). Data are presented as mean ±SEM and Student's *t*‐test was conducted to compare data between two groups. BH‐adjusted *p* values: *p < 0.05; **p < 0.01; ***p < 0.001. G) Performance of the RF model in the test cohort of 29 suspected NS patients (Cohort 3). A scatter plot of the predicted values against the average molecular intensity is shown in the left panel, and a scatter plot of the predicted values against the ELISA results is shown in the right panel. Patients labeled in red were predicted to have NS; the other patients labeled in orange were predicted to have NNS.

To further identify reliable NS biomarkers, we constructed a scoring table derived from machine learning model scores, fold changes, and statistical *p* values between the NS group and the other groups (Figure S4A, Supporting Information). Based on this scoring table, the top 80 proteins identified were subjected to PRM‐MS verification. Next, we collected 131 CSF samples, including 35 NS, 14 IBD, 32 NIBD, 11 PTNS samples, and 39 control group samples (NNS), as an independent cohort (Cohort 7, Figure 3A); 58 of the 80 proteins were identified by PRM as potential biomarkers (Figure S4B; Table S4, Supporting Information), with expression patterns aligning with previous findings from Cohorts 1, 5, and 6 (Figure 1B, Figure 3B). Most of the upregulated NS proteins correlated positively with clinical indicators for diagnosing NS (**Figure** **4**A), such as inter‐alpha‐trypsin inhibitor family members (ITIH1, ITIH2 and ITIH4), which act as carriers of hyaluronan; serpin family A member 3 (SERPINA3), which can inhibit neutrophil cathepsin G; and mast cell chymase. In contrast, most of the downregulated proteins in the NS cohort correlated negatively with clinical indicators (Figure 4B). This group comprises the important negative regulator of oligodendrocyte differentiation and axonal myelination (LINGO1), the serine proteinase inhibitor that regulates axonal growth (SERPINI1), a presynaptic protein that is involved in dopaminergic synaptic transmission (CNTNAP4), and SEMA7A.

Additionally, 21 upregulated proteins and 37 downregulated proteins, such as SEMA7A, SERPINA3, ITIH4, LINGO1, SERPINI1, CNTNAP4, ITIH1, and ITIH2, were detected in the PTNS group according to PRM results as well as DIA (Figure 4C,D; Figure S5B,C, Supporting Information). Pathology revealed that compared with those in the normal control (NC) group, expression levels of LINGO1, SERPINI1, CNTNAP4, and SEMA7A were decreased in NS brain tissue, and expression levels of ITIH1, ITIH4, and SERPINA3 were increased (Figure 4E), indicating the potential utility of these genes in distinguishing the NS group from the NNS group. Notably, SEMA7A, a membrane‐anchored protein, is known to promote neuronal axon outgrowth and regulate synapse formation and elimination in the nervous system. This protein is widely expressed in multiple brain regions, including the hippocampus, hypothalamic‐pituitary system, mesodiencephalic dopamine system, and spinal cord.^[^ ^16^, ^17^ ^]^ Therefore, insufficient SEMA7A in the CSF or brain tissues of NS patients may suggest neuronal damage. ITIH4, a constituent of the inter‐alpha inhibitor protein (IAIP) family, is recognized as an indication of neuroinflammation and is instrumental in assessing the extent of spinal cord injury damage.^[^ ^18^, ^19^ ^]^ This protein is modulated by IL‐6 and is highly expressed during the acute phase of the inflammatory response, independent of the proinflammatory factor TNF‐α.^[^ ^20^ ^]^ SERPINA3 has been identified as a candidate astrocyte factor contributing to blood‒brain barrier (BBB) dysfunction.^[^ ^21^ ^]^ In a co‐culture model derived from human induced pluripotent stem cells for the BBB, SERPINA3 expression was found to increase upon activation by TNF, transitioning astrocytes into an inflammatory reactive state, thereby leading to BBB dysfunction.^[^ ^22^ ^]^ Expression levels of ITIH4 and SERPINA3 are associated with CNS inflammation and predict the severity of encephalitis in NS patients.

To verify the reliability of these potential biomarkers, we expanded the population and collected an additional 89 CSF samples (44 NS and 45 NNS) for ELISA verification (Cohort 8, Figure 3A). We observed significantly upregulated expression of ITIH4 and SERPINA3 and downregulated expression of SEMA7A in the NS group compared to the NNS group (Figure 4F). We also tested CSF from PTNS patients and found expression levels of ITIH4 and SERPINA3 to be decreased (Cohort 9, Figure 3A; Figure S5A, Supporting Information). These results offer additional support for the potential of these three proteins as diagnostic markers for NS.

Next, we evaluated the performance of our scoring model in clinical diagnosis of NS by analyzing 29 CSF samples from SNS patients who did not meet the CDC's diagnostic criteria for NS (Cohort 3, Figure 2A). The scoring system classified 19 patients as likely NS patients and 10 as likely NNS patients. This classification was substantiated by the phenomenon that the patients with high predicted NS scores exhibited symptoms such as memory decline (S‐24), disturbance of consciousness (S‐4, S‐8), and decreased binocular vision (S‐8, S‐10) (Figure 4G). Additionally, we selected one of the biomarkers, ITIH4, for verification (Cohort 9, Figure 3A; Figure S6B, Supporting Information) and found strong concordance between the model predictions and ELISA results (Figure 4H). ITIH4 expression was upregulated in the CSF of S‐8 and S‐10 samples. Together, our scoring model and identified biomarkers showed that patients with high NS scores should receive close clinical monitoring.

## 3 Discussion

Diagnosis and management of NS have long posed significant challenges to medical practitioners. Invasion of the CNS by *T. pallidum* can manifest as various symptoms, such as meningitis, blurry vision, cognitive impairment, and psychiatric symptoms. Termed “the great imitator”, accurate diagnosis of NS cannot rely solely on clinical symptoms or physical signs; laboratory diagnostics play a critical role. CSF evaluation is central to NS diagnosis, though no universal consensus exists on its diagnostic criteria. Commonly observed laboratory abnormalities in NS patients include pleocytosis and increased protein concentrations, but these CSF changes are not specific to NS. Moreover, the VDRL and RPR assays, traditionally considered benchmarks for specificity, are limited by low sensitivity. Therefore, the possibility of NS cannot be ruled out in the event of a negative result.^[^ ^4^, ^23^, ^24^ ^]^ This limitation underscores the suboptimal performance of current diagnostic tools. In fact, none of the currently available tests represent an ideal, universally applicable gold standard, nor is there consensus on the diagnostic criteria for NS.^[^ ^25^ ^]^

Several studies have investigated potential CSF biomarkers in NS patients using protein microarray,^[^ ^26^, ^27^ ^]^ metabolomics analysis,^[^ ^28^, ^29^ ^]^ ELISA,^[^ ^30^ ^]^ and reverse transcription polymerase chain reaction MicroRNAs (RT‒PCR‐miRNA) methods.^[^ ^31^ ^]^ Those studies revealed significantly increased expression levels of various potential biomarkers, including CXCL24, CXCL7, CXCL10, CXCL8, Th1 cytokines (IL‐2, IL‐12, and IFN‐γ), Th2 cytokines (IL‐6 and IL‐10), urokinase plasminogen activator (uPA), soluble triggering receptor expressed on myeloid cells 2 (sTREM2), *N*‐acetyl‐l‐tyrosine, miR‐590‐5p, and miR‐570‐3p.^[^ ^26^, ^27^, ^28^, ^29^, ^30^, ^31^ ^]^ For example, several recent studies have indicated that CSF chemokine ligand 13 (CXCL13) can serve as a supplementary potential diagnostic marker of NS.^[^ ^7^ ^]^ However, those studies investigated relatively small, heterogeneous cohorts, leading to inconsistent sensitivity, specificity, and cutoff values.^[^ ^32^, ^33^ ^]^ In addition, the diagnostic value of these identified biomarkers has not been validated on a large scale. Furthermore, despite contributing valuable insights, these biomarkers are limited by issues of specificity, as their expression is not exclusive to NS and may be indicative of other neurological conditions. For example, CXCL13, a highlighted potential diagnostic marker, is also elevated in Lyme neuroborreliosis, multiple sclerosis, and other inflammatory CNS disorders, thus complicating its diagnostic utility for NS.^[^ ^30^, ^32^, ^34^, ^35^ ^]^ Similarly, Th1 and Th2 cytokines, which are involved in the immune response, can be upregulated in a variety of infectious and autoimmune diseases, detracting from their specificity. The same lack of specificity applies to other biomarkers, such as CXCL10, which is commonly associated with viral infections.^[^ ^22^, ^36^ ^]^ Furthermore, uPA and sTREM2 have been, respectively, implicated in CNS malignancies and neurodegenerative diseases such as neuroblastoma and Alzheimer's disease,^[^ ^37^, ^38^, ^39^ ^]^ which may lead to false‐positive results if used as standalone indicators of NS. The microRNAs miR‐590‐5p and miR‐570‐3p, while novel in their application, have been associated with a variety of pathological processes, including cancer and other infectious diseases,^[^ ^40^, ^41^, ^42^, ^43^ ^]^ thereby raising concerns about their diagnostic specificity for NS.

Our identification of SEMA7A, SERPINA3, and ITIH4 as NS biomarkers is a significant advancement in the search for reliable diagnostic tools. This progress is underscored by our methodological approach, which meticulously contrasted the CSF profiles of NS patients with those of individuals affected by other neurological and infectious brain diseases, as well as syphilis patients without neurological involvement. By conducting such a comprehensive comparative analysis, we enhanced the specificity of the biomarkers we identified, ensuring that they are truly indicative of NS pathology rather than of general inflammatory and/or infectious processes.

The robustness of our findings is further strengthened by the fact that our initial discoveries were corroborated using different methodological approaches. This multipronged validation process not only reinforces the reliability of SEMA7A, SERPINA3, and ITIH4 as biomarkers but also suggests that they possess a strong discriminatory capacity to distinguish NS from other conditions. Such differentiation is crucial for clinicians who are tasked with making accurate diagnoses in the presence of overlapping clinical presentations.

The specificity of our biomarkers is particularly noteworthy. For example, SEMA7A has not been widely reported in other CNS diseases, making it a compelling candidate for NS‐specific pathology. SERPINA3 and ITIH4 also exhibited unique expression patterns in our NS CSF samples, which was not detected in samples from our patients of other neurological cohorts. This level of specificity is paramount in the context of NS, in which misdiagnosis can lead to inadequate treatment and potentially severe outcomes.

In this study, we analyzed 223 CSF samples to identify promising biomarkers for diagnosing NS. Furthermore, we constructed a machine learning model‐based scoring table. This model, which integrates a scoring matrix from a machine learning classifier with proteomics profiling features, was applied to interpret “suspected” samples and aid in diagnosing NS. The biomarkers of these “confirmed” NS patients were simultaneously interpreted, and the patients were followed up to verify the reliability of the biomarkers. Using multicenter ELISA validations, we selected three biomarkers (SEMA7A, SERPINA3, and ITIH4) and confirmed their efficacy in distinguishing NS from syphilis and other brain diseases, including infectious diseases.

One limitation of our study is the emphasis on identifying potential biomarkers for diagnosis of NS but not for reflecting its severity, which is an aspect that warrants further exploration. Additionally, we recognize that establishing a case definition for NS presents another challenge of our study, given the lack of widely accepted diagnostic criteria for this clinical condition. Additionally, although the current study provides valuable insights through CSF proteomic analysis, we acknowledge the greater potential of comparing these findings with proteomic data from neuropathological brain samples. Such a comparative approach might significantly strengthen our conclusions and offer a more comprehensive understanding of NS and recommend this methodology as a meaningful next step for future research to achieve more robust and compelling results on this topic.

## 4 Conclusion

In conclusion, a valuable diagnostic tool for NS is critical in management of this disease and might prevent further disease progression with timely treatment. Our study identified SEMA7A, SERPINA3, and ITIH4 in the CSF as candidate biomarkers for NS diagnosis with high accuracy, which was confirmed in the brain tissues of NS patients. Importantly, a significant constraint in the current diagnostic approaches for NS lies in the limited ability to further differentiate suspected NS patients and confirm their CNS status. The discovered biomarkers will be instrumental in determining whether suspected cases are indeed cases of NS. Therefore, this tool will serve as an important complement for diagnosing NS rather than as a replacement for clinical diagnostic criteria. Investigations are actively ongoing to unravel the regulatory mechanisms underlying expression of these biomarkers and the pathogenesis of the brain parenchyma damage caused by *T. pallidum*.

## 5 Experimental Section

### Ethics Approval and Patient Consent Statement

This study was approved by the ethics committee of Peking Union Medical College Hospital (PUMCH, Approval ID: ZS‐1754, ZS‐1105) and Beijing You'an Hospital (Approval ID: LL‐2019‐039‐K). All participants provided written informed consent before CSF sample collection through lumbar puncture under protocols approved by the Institutional Review Board at PUMCH and Beijing You'an Hospital.

### Study Design and Case Definition

This study included six distinct patient groups: 1) NS patients; 2) NNS syphilis patients; 3) SNS patients; 4) PTNS patients; 5) patients with IBD unrelated to syphilis; and 6) NIBD patients without syphilis. The diagnostic criteria for NS were based on positive results of a RPR test and a particle agglutination assay for antibodies against *Treponema pallidum* (TPPA) in CSF. NNS was characterized by negative CSF RPR and TPPA results in syphilis patients without CSF pleocytosis (>5/µL) and elevated CSF protein levels (>45 mg dL^−1^) in the absence of any characteristic symptoms or signs consistent with NS. Syphilis patients with negative CSF RPRs but CSF pleocytosis and/or elevated CSF protein levels, along with clinical symptoms or signs consistent with NS without other known causes, were considered to have SNS. Patients with PTNS were defined as patients with confirmed NS who received the gold standard treatment (i.e., intravenous benzylpenicillin, 24 million international units per day for 10–14 days; alternatively, ceftriaxone, 1–2 g intravenously daily for 10–14 days), after which their RPR titer decreased at least fourfold or recovered to normal after 6 months. Patients in the syphilis‐free group were identified among those who visited the hospital due to neurological symptoms and were ultimately diagnosed with other CNS diseases, encompassing both infectious and noninfectious brain diseases. The exclusion criteria included traumatic CSF collection, therapeutic intervention (except for serofast or PTNS group participants), and pregnancy. Sociodemographic and clinical characteristics (including clinical information and laboratory test data) were extracted for analysis.

Sociodemographic information included sex, age, and ethnicity. Clinical data included diagnosis, symptoms presented at the time of diagnosis, and treatment. Laboratory results for syphilis included the following results: RPR (Shanghai Kehua Bioengineering Co., Ltd., China), TPPA (Fujirebio Inc., Japan), and fluorescent treponemal antibody absorption (EUROIMMUN AG, Inc., Germany). All patient metadata, including age, sex, and diagnostic information for NS, are presented in Table S1, Supporting Information.

### Cerebrospinal Fluid Sample Collection

CSF samples were collected and stored in accordance with international guidelines.^[^ ^44^ ^]^ The puncture locations were between the lumbar vertebrae L3/L4, L4/L5, or L5/S1, and the CSF samples were collected into sterile polypropylene tubes, divided into 0.5 mL aliquots, and frozen at −80 °C within 1 h.

### Brain Tissue Samples and Case Classification

Brain tissue samples were obtained from paraffin sections from the Pathology Department of PUMCH. The tissues were collected from the frontal and temporal lobes. Warthin–Starry staining revealed spirochetes in the brain tissue samples of patients with NS.^[^ ^45^ ^]^ The tissue samples of the standard control group were taken from the postmortem tissue of syphilis‐free patients without any encephalopathy. Brain tumor and demyelinating disease tissues was used from patients in the NIBD group and brain abscess, cryptococcus, or other infection tissues from patients in the IBD group. All patient metadata, including age, sex, and diagnostic information for NS, are provided in Table S1 (Supporting Information).

### Mass Spectrometry and Data Processing

Proteins were extracted from CSF samples and then processed and digested into peptide mixtures. The peptide mixtures were further analyzed using an Orbitrap Fusion mass spectrometer equipped with an Easy‐nLC 1000 nanoflow liquid chromatography system by data‐dependent acquisition (DDA) or DIA strategy. Proteome Discoverer (version 2.3.0.523) was used to analyze the DIA data. Then, all DDA data were loaded into Spectronaut (v.14.10.201222.47784) to generate a sample‐specific spectral library; the raw DIA data were processed using Spectronaut with default settings. Detailed information about the proteomic technique and data analysis is provided in the Supplementary Methods.

### Random Forest‐based Machine Learning Model

Based on the proteomic data of Cohorts 1 and 2 (Table S1, Supporting Information), we developed a new RF‐based classifier to identify potential biomarkers to classify NS patients (Figure 1D). Development of a computational pipeline involves the following five steps: i) dataset preprocessing, ii) feature selection, iii) model training, iv) identification of the most important features, and v) model evaluation.

Data from NNS and NS patient samples (Cohorts 1, 2, and 3) were log2‐transformed and mean‐centered. The R package caret^[^ ^46^ ^]^ (v.6.0‐90) was subsequently used to sample 70% of Cohort 1 (DIA) (NNS, *n* = 25; NS, *n* = 15) as a training set, whereas the remaining samples of Cohort 1 were used as an independent testing set.

To eliminate redundant features and noise, we first selected differentially expressed proteins (BH adjusted *p* value < 0.01, 358 proteins; Table S2, Supporting Information) between the NS and NNS samples to construct the RF model. Parameters were tuned using a grid search algorithm with threefold cross‐validation implemented in the caret package. After model fitting, features were ranked according to importance, retaining those with a mean decrease in accuracy greater than 0.5. Finally, the top 80 important features were retained for PRM‐MS verification according to a composite score system based on fold change, statistical significance, and mean decrease accuracy. Furthermore, the RF model was retrained using the same strategy based on 58 of the 80 features verified via PRM‐MS. The training set consisted of 70% of the samples, and the evaluation was conducted using the remaining 30% of the independent testing set from Cohort 2 (PRM‐MS). The performance of the RF model was further assessed based on an independent test set from Cohort 3 (PRM‐MS). Corresponding confusion matrices and ROC plots were generated for assessing the performance of the assemblers using the caret, plotROC^[^ ^47^ ^]^ (v.2.2.1), and ggplot2 packages.

### Enzyme‐linked Immunosorbent Assay

Human protein enzyme‐linked immunosorbent assay kits were used to quantify CSF levels of endogenous proteins according to the manufacturer's protocols. Briefly, CSF samples were diluted for detection of SEMA7A (1:1), ITIH4 (1:100), and SERPINA3 (1:200). Fixed dilutions of CSF samples (100 µL) were added to precoated plates, and the plates were incubated at 37 °C for 2 h. After washing, 100 µL of detection antibody was added to each well, and the plates were again incubated at 37 °C for 2 h. Following another wash, 100 µL of the working dilution of streptavidin‐HRP was added to each well. The plate was covered and incubated for 20 minutes at room temperature in the dark. Finally, optical density was determined at 450 nm after adding 50 µL of stop solution.

### Statistical Analysis

All data analyses and visualizations were performed using R package (version 4.0.3). Protein expression values were quantified, log2‐transformed and mean‐centered. Multiple comparisons among the NS, NNS, PTNS, NIBD, and IBD patients were performed using the R package Limma (v.3.38.3) to test for significant differences in expression of proteins. Pairwise comparisons were also carried out to determine the proteins with significantly different expression levels between pairs within the five experimental groups using Limma. Differences with a BH adjusted *p* value ≤ 0.01 were considered to indicate statistical significance. The statistical data of protein intensities are presented as means ± S.E.M.s. Student's *t*‐test was conducted using GraphPad Prism (version 9.0.0) to compare data between two groups. Each experiment was performed at least three times (*n*≥3), and the specific sample size was provided in the legends of figures. The results were considered significant at *p* ≤ ^***^0.001, ^**^0.01, or ^*^0.05.

The online tool DAVID (https://david.ncifcrf.gov/)^[^ ^48^ ^]^ was used to annotate proteins according to biological process, cellular component, and molecular function terms via Gene Ontology^[^ ^49^ ^]^ analysis. Moreover, this tool assessed enrichment of biological pathways associated with differentially expressed proteins via KEGG^[^ ^50^ ^]^ pathway analysis. A global protein interactome network was constructed for proteins with differential expression between groups using Cytoscape (v.3.8.2),^[^ ^51^ ^]^ and protein–protein interactions were retrieved from the STRING database.^[^ ^52^ ^]^ Principal coordinate analysis of proteins with valid values in each sample was performed using the R package ape.^[^ ^53^ ^]^ Volcano plots and heatmaps were generated to visualize the quantified values for proteins that displayed significant differences in expression between groups using the R packages ggplot2 and ComplexHeatmap^[^ ^54^ ^]^ (distance: Pearson; linkage: complete).

## Conflict of Interest

The authors declare no conflict of interest.

## Author Contributions

Jun Li, J.M., Mingjuan Liu, Mansheng Li, M.Z., W.Y., and M.W. contributed equally to this work. L.L., Jun Li, and J.M. conceived the overall study and designed experiments. L.L., Jun Li, and Y.Z. had full access to all the data in the study and took responsibility for the data analysis accuracy. Jun Li, Mingjuan Liu, M.Z., W.Y., M.W., Hanlin Zhang, Heyi Zheng, C.M., J.S., Wenze Wang, W.L., X.Y., Wenjia Weng, Juan Li, and F.C. performed the CSF and brain tissue samples preparation and clinical information collection. J.M., Mansheng Li, L.L., and Y.Z. performed proteomics experiments and data analysis. J.M., Mansheng Li, and X.L. performed the statistical and bioinformatics analysis. L.L., Mingjuan Liu, and Q.Z. performed most biological and functional experiments. L.L., J.M., Jun Li, Mingjuan Liu, Mansheng Li, and Y.Z. wrote and edited the manuscript. Jun Li, L.L., and Y.Z. provided funding support. All authors made important comments to the manuscript.

## Supporting information

Supporting Information

Supporting Information

Supporting Information

Supporting Information

Supporting Information

Supporting Information

## Data Availability

All the proteomics data generated in this study have been deposited in ProteomeXchange Consortium via the iProX^[^ ^55^, ^56^ ^]^ repository with the identifier PXD033034. The equipment, reagents, and supplies are available in Table S5, Supporting Information.
