# Supplementary material for: Large‐Scale Proteome Profiling Identifies Biomarkers Associated with Suspected Neurosyphilis Diagnosis
Source: Adv Sci (Weinh). 2024 Feb 21;11(16):2307744. doi: 10.1002/advs.202307744 (PMC11040343; doi:10.1002/advs.202307744)
Supplement: Supplementary file 1 — Supporting Information [file ADVS-11-2307744-s004.pdf]

ADVANCED SCIENCE
Open Access

Supporting Information

for *Adv. Sci.*, DOI 10.1002/advs.202307744

Large-Scale Proteome Profiling Identifies Biomarkers Associated with Suspected Neurosyphilis Diagnosis

Jun Li, Jie Ma, MingJuan Liu, Mansheng Li, Ming Zhang, Wenhao Yin, Mengyin Wu, Xiao Li, Qiyu Zhang, Hanlin Zhang, Heyi Zheng, Chenhui Mao, Jian Sun, Wenze Wang, Wei Lyu, Xueping Yue, Wenjia Weng, Juan Li, Fengxin Chen, Yunping Zhu* and Ling Leng**

Supporting Information

Large-scale Proteome Profiling Identifies Biomarkers Associated with Suspected Neurosyphilis Diagnosis

Jun Li[†], Jie Ma[†], MingJuan Liu[†], Mansheng Li[†], Ming Zhang[†], Wenhao Yin[†], Mengyin Wu[†], Xiao Li, Qiyu Zhang, Hanlin Zhang, Heyi Zheng, Chenhui Mao, Jian Sun, Wenze Wang, Wei Lyu, Xueping Yue, Wenjia Weng, Juan Li, Fengxin Chen[j], Yunping Zhu[,*], Ling Leng[*]*

[†] These authors contributed equally to this work.

* Correspondence: Ling Leng (lengling@pumch.cn) or Jun Li (lijun35@hotmail.com) or Yunping Zhu (zhuyunping@ncpsb.org.cn).

Supplementary methods

Protein extraction and tryptic digestion

Protease inhibitor was added to the CSF samples and a concentrated sample. Then, the supernatants were transferred to new centrifuge tubes. The top 14 high-abundance proteins were removed using High-Select Top 14 Abundant Protein Depletion Resin (Thermo Fisher Scientific). Then, the supernatants were collected and the protein concentrations were determined using a Bradford kit according to the manufacturer's protocols. Next, the samples were transferred into 10-KDa ultrafiltration filters and replaced liquid with UA buffer (8 M urea, 150 mM Tris-HCl, pH 8.0). Then, 25 mM DTT was added to the samples, which were incubated for 1 h at 37°C. After centrifugation at 14,000 × *g* for 15 min at 25°C, 50 mM iodoacetamide was added to the samples, followed by incubation in the dark for 30 min at 25°C. Next, 25 mM NH_4HCO_3 was added to the samples, which were centrifuged for 10 min and repeated four times. Then, final digestion was performed at 37°C overnight by incubating the samples with trypsin (enzyme-to-substrate ratio of 1:50). The samples were washed three times using 100 μL of 25 mM NH_4HCO_3 and centrifuged at 12,000 × *g* for 10 min. Finally, the supernatants containing peptide mixtures were transferred to clean tubes for LC–MS/MS.

Samples from organoids were scraped into new EP tube with 20 μL of urea buffer (8 M urea, 150 mM Tris-HCl, 10 mM DTT, pH 8.0). An additional 10 μL buffer was added to the tube. Steel balls were added to the 30-μL buffer for vibration (70 Hz) for 1 min. After centrifugation at 14,000 × *g* for 10 min at 4°C, the supernatants were transferred to clean tubes. Next, the extracted proteins were reduced at 37°C for 1 h and alkylated in 25 mM iodoacetamide at room temperature for 30 min in the dark. Finally, the protein samples were digested with Lys C (1 μg at

37°C for 4 h) and trypsin (enzyme-to-substrate ratio of 1:50) at 37°C for 16 h, desalted using C18 cartridges and vacuum-dried using a Speed Vac.

High pH reversed-phase chromatography

The digests were further fractionated using high pH reversed-phase chromatography for data-dependent acquisition (DDA) samples. One hundred micrograms of the digest were combined. A reverse chromatography column was used for separating the mixed peptides and performed by a RIGOL L-3000 system (RIGOL, Beijing, China). The peptide mixtures were dissolved in 100 μL mobile phase A (2% [v/v] acetonitrile, 98% [v/v] ddH$_2$O, pH 10) and then centrifuged at 14,000 × *g* for 20 min. The supernatants were loaded into XBridge peptide BEH C18 columns (130Å, 3.5 μm, 4.6 mm × 150 mm; Waters Corp) and eluted stepwise by injecting the mobile B (98% [v/v] acetonitrile, 2% [v/v] ddH$_2$O, pH 10). The flow rate was set at 1 mL/min. The fractions were eluted (1 min each) and collected using step gradients of mobile phase B. Forty fractions were collected along with the LC separation, which was subsequently pooled (in a nonsequential fashion) into 10 fractions. The final 10 fractions were freeze-dried and stored at −80°C.

Mass spectrometry (MS) analysis

The peptide mixtures were analyzed using an Orbitrap Fusion mass spectrometer equipped with an Easy-nLC 1000 nanoflow liquid chromatography system. After drying, the peptides were resuspended in 0.1% formic acid and loaded onto a reverse chromatography column (75 μm × 25 mm, 1.9 μm; Thermo Fisher Scientific). For the proteome profiling samples, peptides were separated on an analytical column over a 90-min gradient (buffer A: 0.1% formic acid and 99.9% H$_2$O; buffer B: 0.1% formic acid and 99.9% acetonitrile at a constant flow rate of 0.3 μL/min (0–4 min, 3%–8% buffer B; 4–69 min, 8%–22% buffer B; 69–81 min, 22%–35% buffer B; 81–85 min,

35%–90% buffer B; 85–90 min, 90% buffer B).

For DDA–MS runs, the entire MS scanning range was from 300 to 1500 m/z. The resolution for MS was set to 60,000 and then under 2.5-s top speed mode for 15,000-resolution MS/MS scans. For high-energy collision dissociation, the isolation window was set to 1.6 m/z, and a normalized collision energy of 32% was applied. For DIA–MS runs, the entire MS scan was from 300 to 1500 m/z. Then, DIA segments were acquired at the resolution of 30,000, and the collision energy was 33%. The spectra were recorded in profile mode. The default charge state for the MS2 was set to 3.

Based on the results of the DIA experiment, we selected the top 58 proteins for Parallel Reaction Monitoring-Mass Spectrometry (PRM-MS) verification according to the scoring table. For PRM-MS analysis, the peptide mixtures were analyzed using an Orbitrap Fusion mass spectrometer equipped with an Easy-nLC 1000 nanoflow liquid chromatography system. For the proteome profiling samples, the peptides were separated on a home-made analytical column (150 μm × 150 mm, 1.9 μm) over a 60 min gradient (buffer A: 0.1% formic acid and 99.9% H_2O; buffer B: 0.1% formic acid, 80% acetonitrile and 19.9% H_2O) at a constant flow rate of 0.5 μL/min (0–2 min, 6%–10% buffer B; 2–40 min, 10%–22% buffer B; 40–48 min, 22%–35% buffer B; 48–55 min, 35%–90% buffer B; 55–60 min, 90% buffer B). For the full mass spectrometry survey scan, the resolution was 60,000, the scan ranged from 300 to 1,250 m/z, the AGC target was 1×10^6, and the max IT was 50 ms. For the MS2 scan, the resolution was 30,000; charge state screening was enabled (to retain precursor ions containing a charge between +2 and +6).

Proteomic MS/MS data processing

The Proteome Discoverer software (version 2.3.0.523; Thermo Fisher Scientific) were

used to analyze the DDA data and search against the UniProt human database (downloaded on 2019-7-31, containing 73,940 proteins). The parameters used for database searches were as set follows: precursor and fragment mass tolerances of 10 ppm and 0.02 Da, respectively; trypsin as the digestion enzyme; a maximum number of missed cleavage sites of 2; oxidation (M) and acetylation (protein N-terminus) set as dynamic modifications; and carbamidomethylation of cysteine set as a fixed modification. The identified proteins were filtered at both the peptide and protein levels at a 1% false discovery rate, determined by a target-decoy search strategy. All DDA results were loaded into Spectronaut (v.14.10.201222.47784; Biognosys, Switzerland) to generate the sample-specific spectral library. Then, the raw DIA data were processed on Spectronaut using the default settings. Briefly, the retention time prediction type was set to dynamic iRT and correction factor for the window. Mass calibration was set to local mass calibration. Decoy generation was set to scramble (no decoy limit). Removing fragments for quantification based on interfering signals, the interference correction on the MS2 level was enabled, maintaining at least three fragments for quantification. The false discovery rate was estimated with the mProphet approach and set to 1% at the peptide level. Protein inference was performed on the principle of parsimony using the ID Picker algorithm implemented in Spectronaut. The RAW files were converted into the Spectronaut file format to analyze the DIA runs with the spectral library. The files were then calibrated using the global spectral library in the retention time dimension. Subsequently, the recalibrated files were used for targeted data analysis with the spectral library without new recalibration of the retention time dimension.

A spectral library from PRM-MS analysis was also constructed from the DDA data, and unique peptides of the target proteins were selected and exported to set the

PRM-MS method. First, the raw MS files from the PRM-MS data were processed in Skyline (v.20.1.0.155). Next, the library's top five product ions of target proteins were used for comparison and quantification. The data were deemed reliable when the peak shape was intact and the retention time was within the set retention time range, and the undetected product ions were manually removed. Then, the peptide peak areas observed in samples from patients were exported into Excel for further analysis.

Supplementary Figures

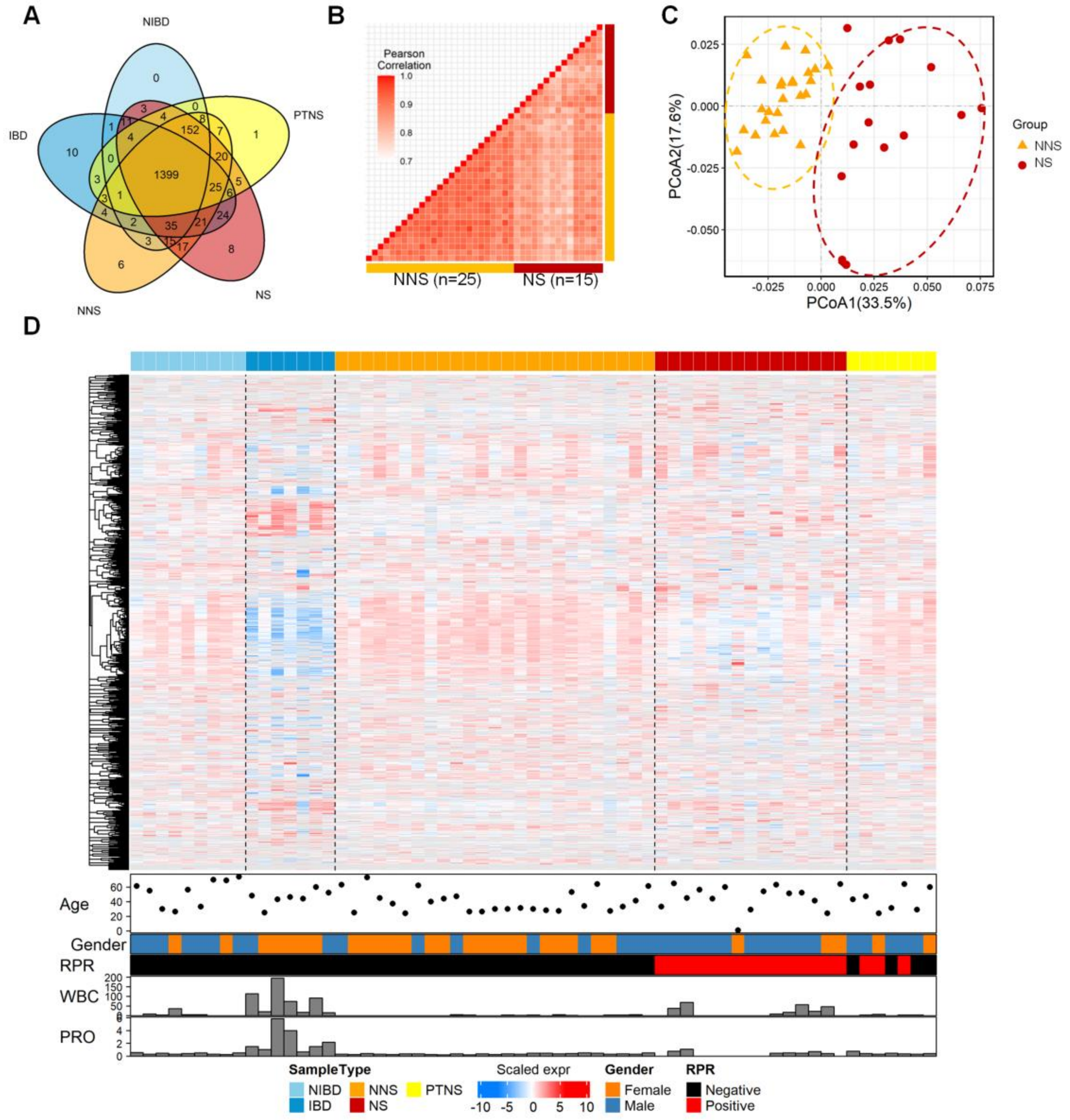

Figure S1. Proteomic profile of CSF samples from NNS, NS, PTNS, IBD, and NIBD patients using quantitative proteomics based on DIA technology. A) Venn diagram of protein identified in CSF from NNS, NS, PTNS, IBD, and NIBD samples. B) Heatmap of overall relatedness of NNS and NS groups. The map plots the pairwise Pearson's correlation coefficients derived from NNS and NS samples by the pattern of relative protein abundance (range: 0.85–1.00). C) PCoA analysis of the proteome profile of NS (n = 15) and NNS samples (n = 25). D) Consensus-clustering analysis of proteomic profiling within five proteomic groups: NNS (yellow, n = 25), NS (red, n =

15), PTNS (yellow, n = 7), IBD (light blue, n=7), and NIBD (dark blue, n = 9). The associations of five proteomic groups with clinical characteristics (CSF-RPR, CSF-WBC, CSF-PRO) are annotated in the below panel. PRP: rapid plasma reagin, WBC: white blood cells, PRO: protein. The red and blue scale represents the scaled expression levels of proteins among these groups. NNS: syphilis/non-neurosyphilis; NS: neurosyphilis; PTNS: post-treatment neurosyphilis patients; NIBD: syphilis-free but with noninfectious brain disease; IBD: syphilis-free but with infectious brain disease.

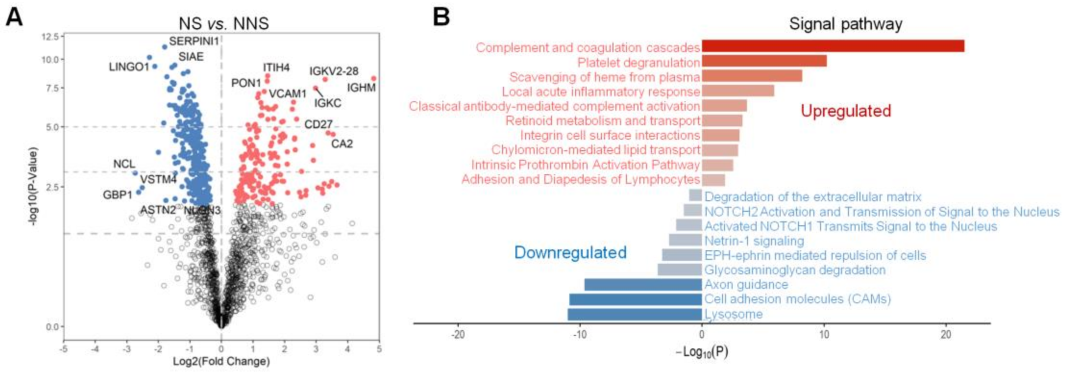

Figure S2. Analysis of differentially expressed proteins of CSF samples between NS and NNS patients. A) Volcano plots of –log10 p-value vs. log2 protein abundance comparisons for CSF between NS and NNS groups. Pairwise comparisons are carried out using Limma to determine the proteins with significantly different expression levels. Proteins outside the significance threshold lines (−log10 (p-value) >2 and |log2 (NS/NNS)| >1) are in red (upregulated) or blue (downregulated). B) Signal pathway analysis of the differentially expressed proteins between NS and NNS samples based on the enrichment values (-Log10 (p-value)). Red and blue columns represent the up- and downregulated proteins of NS vs. NNS groups. NNS: syphilis/non-neurosyphilis; NS: neurosyphilis.

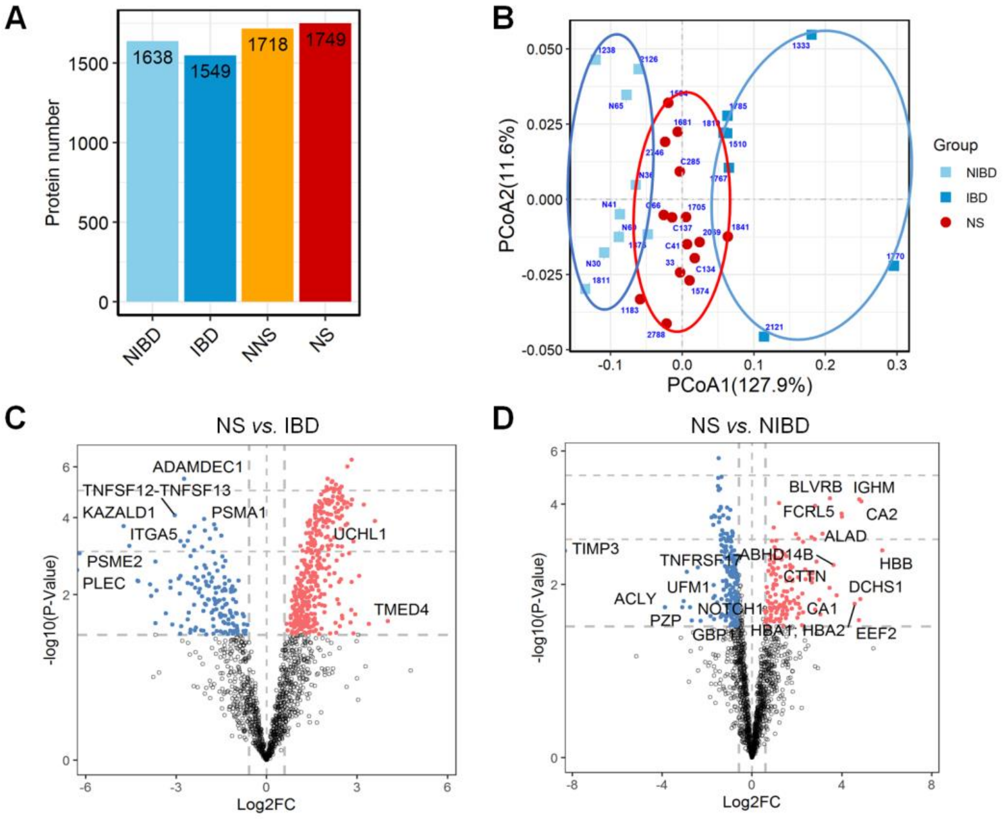

Figure S3. Proteomic features of CSF samples from NNS, NS, IBD, and NIBD patients. A) Distribution of protein numbers identified in CSF from NNS, NS, IBD, and NIBD samples. B) PCoA analysis of the proteome profile of NS (n = 15), IBD (n=7) and NIBD samples (n = 9). Volcano plots of–log10 p-value vs. log2 protein abundance comparisons for CSF between NS and IBD (C) or NIBD (D) groups. Pairwise comparisons are carried out using Limma to determine the proteins with significantly different expression levels. Proteins outside the significance threshold lines (−log10 (p-value) > 2 and |log2 (NS/IBD or NIBD)| > 1) are in red (upregulated) or blue (downregulated).

A

Terms	Description	A	B	C	D
RF-based Score	Mean Decrease Accuracy	Score≥2	2>Score≥1	1>Score≥0	Score<0
Fold Change (NS/NNS)	log_2NS/NNS	$\|log_2FC\| \geq 1$	$1 > \|log_2FC\| \geq 0.585$	$0.585 > \|log_2FC\| \geq 0.263$	$\|log_2FC\| < 0.263$
P-value (NS/NNS)		p≤0.001	0.001>p≥0.01	0.011>p≥0.05	p>0.05
Fold Change (PTNS/NS)	log_2PTNS/NS	$\|log_2FC\| \geq 1$	$1 > \|log_2FC\| \geq 0.585$	$0.585 > \|log_2FC\| \geq 0.263$	$\|log_2FC\| < 0.263$
P-value (PTNS/NS)		p≤0.001	0.001>p≥0.01	0.011>p≥0.05	p>0.05
Fold Change (NNS/IBD)	log_2NC/IBD	$\|log_2FC\| \geq 1$	$1 > \|log_2FC\| \geq 0.585$	$0.585 > \|log_2FC\| \geq 0.263$	$\|log_2FC\| < 0.263$
P-value (NNS/IBD)		p≤0.001	0.001>p≥0.01	0.011>p≥0.05	p>0.05
Fold Change (NS/NIBD)	log_2NS/NIBD	$\|log_2FC\| \geq 1$	$1 > \|log_2FC\| \geq 0.585$	$0.585 > \|log_2FC\| \geq 0.263$	$\|log_2FC\| < 0.263$
P-value (NS/NIBD)		p≤0.001	0.001>p≥0.01	0.011>p≥0.05	p>0.05
Fold Change (NS/IBD)	log_2NSP/IBD	$\|log_2FC\| \geq 1$	$1 > \|log_2FC\| \geq 0.585$	$0.585 > \|log_2FC\| \geq 0.263$	$\|log_2FC\| < 0.263$
P-value (NS/IBD)		p≤0.001	0.001>p≥0.01	0.011>p≥0.05	p>0.05

B

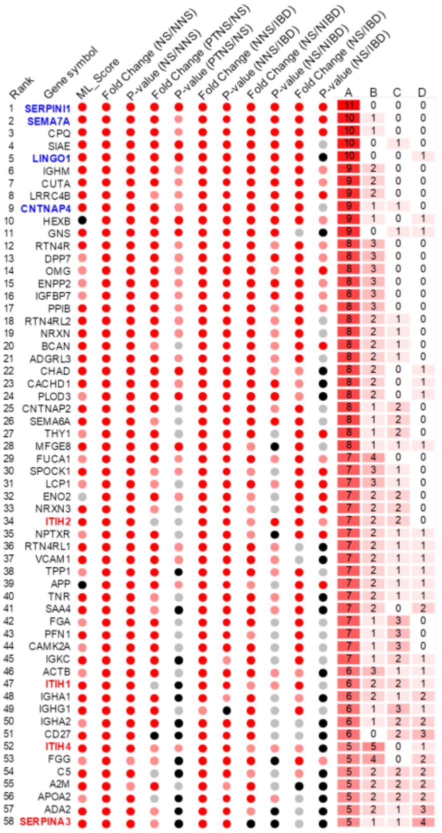

Figure S4. Construction of scoring table for potential biomarkers based on proteomic features and machine learning model scores. A) A score table for the selection of PRM-MS verification proteins, including 11 detailed scoring terms. The description of each item and the specific meaning of the level are provided in this table. B) Visualization of the 58 proteins verified by PRM-MS. Circles of different colors represent each item's grading levels (A, B, C, and D). Red boxes indicate the numbers of each level score (circles of different colors) enriched.

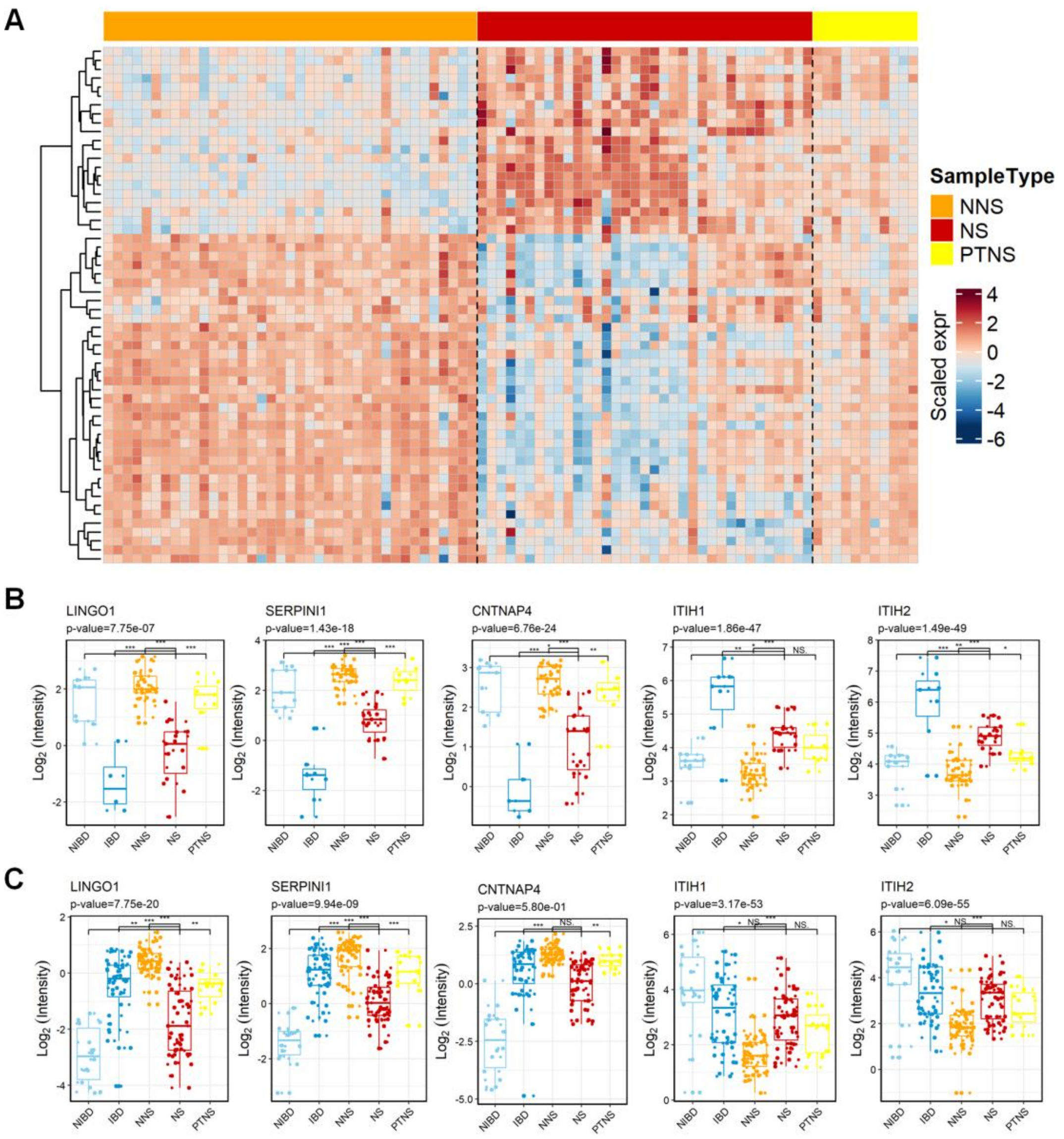

Figure S5. Identification of potential biomarkers that can distinguish NS from PTNS. A) Heatmap showing the PRM-MS verified differentially expressed proteins (totally 58 proteins, z-score-normalized log2-transformed value) in CSF among NNS (n=39), NS (n=35), and PTNS (n=11) samples. Protein expression levels of LINGO1, SERPIN1, CNTNAP4, ITIH1, and ITIH2 in CSF from NS, PTNS, IBD, NIBD, and NNS samples were measured by DIA identification (B) and PRM-MS verification (C) according to the normalized protein intensity. Pairwise comparisons are carried out using Limma to determine the proteins with significantly different expression levels with BH-adjusted *p*-value: *, < 0.05; **, < 0.01; ***, < 0.001.

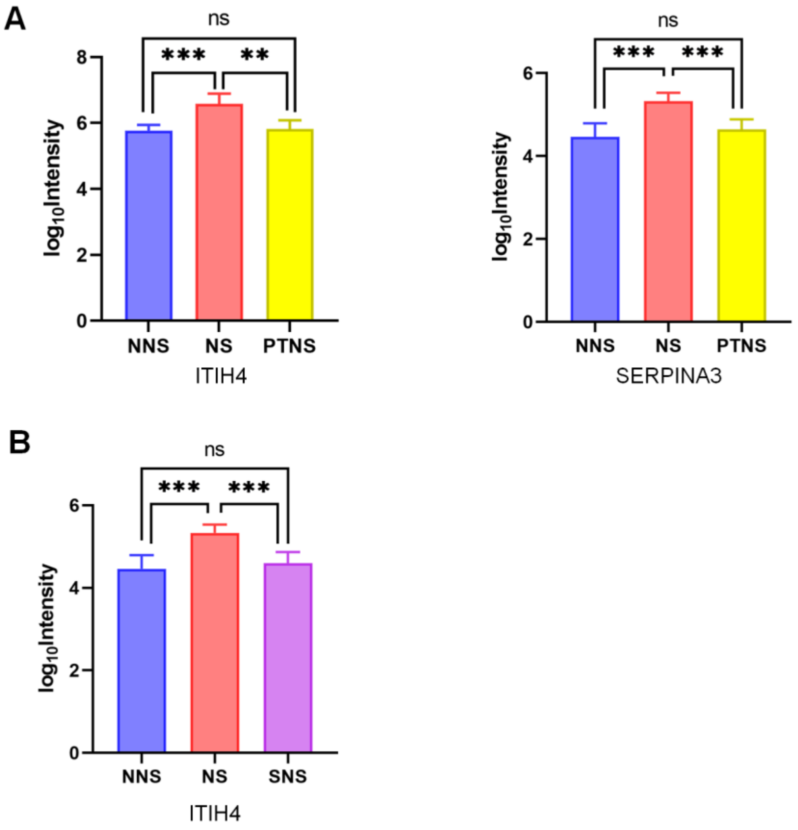

Figure S6. Verification of potential biomarkers in the PTNS and SNS. A) ELISA analysis of ITIH4 and SERPINA3 expression in the CSF of NNS (n = 44), NS (n = 36), and PTNS (n = 11) groups according to Log 10 (intensity). B) ELISA analysis of ITIH4 expression in the CSF of NNS (n = 44), NS (n = 36), and SNS (n = 12) group according to Log 10 (intensity). Data are presented as mean ±SEM and Student's t test was conducted to compare data between two groups (* $p < 0.05$; ** $p < 0.01$; *** $p < 0.001$).
